# 2-[4-(4-Methylphenylsulfonyl)piperazin-1-yl]-1-(4,5,6,7-tetrahydrothieno[3,2-*c*]pyridin-5-yl)ethanone

**DOI:** 10.1107/S1600536811028716

**Published:** 2011-07-23

**Authors:** Duan Niu, Shu-Yun Huang, Ping-Bao Wang, Deng-Ke Liu

**Affiliations:** a Tianjin Medical University, Tianjin 300070, People’s Republic of China; b Tianjin Institute of Pharmaceutical Research, Tianjin, 300193, People’s Republic of China

## Abstract

In the title thienopyridine derivative, C_20_H_25_N_3_O_3_S_2_, the piperazine ring exhibits a chair conformation and the tetra­hydro­pyridine ring exhibits a half-chair conformation. The folded conformation of the mol­ecule is defined by the N—C—C—N torsion angle of −70.20 (2) °. Inter­molecular C—H⋯S and C—H⋯O hydrogen bonds help to establish the packing.

## Related literature

For background to the bioactivity and applications of the title compound, see: Cattaneo (2009 ▶); Wallentin (2009 ▶). For a related structure, see: Zhi *et al.* (2011 ▶). For the synthesis of the title compound, see: Liu *et al.* (2008 ▶).
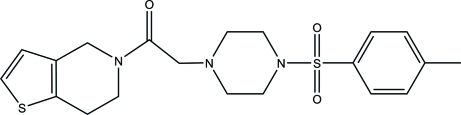


## Experimental

### 

#### Crystal data

C_20_H_25_N_3_O_3_S_2_
*M* *_r_* = 419.55
Orthorhombic, 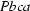

*a* = 13.062 (2) Å
*b* = 15.710 (3) Å
*c* = 19.798 (3) Å
*V* = 4062.8 (11) Å^3^
*Z* = 8
Mo *K*α radiation
μ = 0.29 mm^−1^
*T* = 113 K
0.24 × 0.20 × 0.18 mm

#### Data collection

Rigaku Saturn CCD area-detector diffractometer
Absorption correction: multi-scan (*CrystalClear*; Rigaku/MSC, 2005 ▶) *T*_min_ = 0.934, *T*_max_ = 0.950
49454 measured reflections
4844 independent reflections
4463 reflections with *I* > 2σ(*I*)
*R*_int_ = 0.054

#### Refinement

*R*[*F* ^2^ > 2σ(*F* ^2^)] = 0.054
*wR*(*F* ^2^) = 0.143
*S* = 1.14
4844 reflections
254 parameters
H-atom parameters constrained
Δρ_max_ = 1.17 e Å^−3^
Δρ_min_ = −0.34 e Å^−3^

### 

Data collection: *CrystalClear* (Rigaku/MSC, 2005 ▶); cell refinement: *CrystalClear*; data reduction: *CrystalClear*; program(s) used to solve structure: *SHELXS97* (Sheldrick, 2008 ▶); program(s) used to refine structure: *SHELXL97* (Sheldrick, 2008 ▶); molecular graphics: *SHELXTL* (Sheldrick, 2008 ▶); software used to prepare material for publication: *CrystalStructure* (Rigaku/MSC, 2005 ▶).

## Supplementary Material

Crystal structure: contains datablock(s) global, I. DOI: 10.1107/S1600536811028716/fl2350sup1.cif

Structure factors: contains datablock(s) I. DOI: 10.1107/S1600536811028716/fl2350Isup2.hkl

Supplementary material file. DOI: 10.1107/S1600536811028716/fl2350Isup3.cdx

Supplementary material file. DOI: 10.1107/S1600536811028716/fl2350Isup4.cml

Additional supplementary materials:  crystallographic information; 3D view; checkCIF report

## Figures and Tables

**Table 1:** Hydrogen-bond geometry (Å, °)

*D*—H⋯*A*	*D*—H	H⋯*A*	*D*⋯*A*	*D*—H⋯*A*
C5—H5*A*⋯S1^i^	0.99	2.77	3.469 (2)	128
C6—H6*A*⋯O1^ii^	0.99	2.52	3.470 (3)	161
C6—H6*B*⋯O2^iii^	0.99	2.51	3.346 (3)	143

Symmetry codes: (i) 
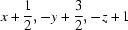
; (ii) 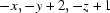; (iii) 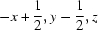.

**Fig. 1.**
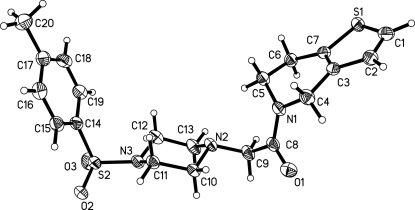
The molecular structure of (I), with the atom-numbering scheme and 50% probability displacement ellipsoids.

**Fig. 2.**
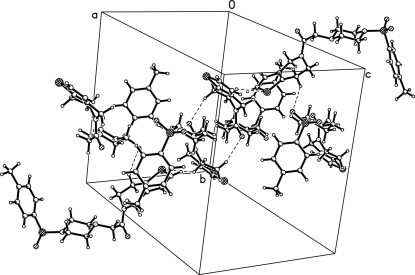
Packing diagram for (I) with hydrogen bonds drawn as dashed lines.

## supplementary crystallographic information

### Comment

As a thienopyridine derivative, the title compound(I) can be used as an irreversible P2Y12 antagonist to inhibit ADP, which induces platelet aggregation and decreases the risk of arterial occlusion. (Cattaneo 2009; Wallentin 2009).

The piperazine ring exhibits a chair conformation and the tetrahydropyridine ring exhibits a half chair conformation (Fig. 1). The folded conformation of the molecule is defined by the N1—C8—C9—N2 torsion angle of -70.20 (2) °. The dihedral angles formed between the tetrahydropyridine plane and the phenyl ring and the C10—C11—C12—C13 plane are 85.47 (6) ° and 56.38 (9) °, respectively. The crystal is stabilized by intermolecular C—H···S and C—H···O hydrogen bonds (Table1, Fig.2).

### Experimental

2-Chloroacetyl chloride was added dropwise into a mixture of 4,5,6,7-tetrahydrothieno[3,2-*c*]pyridine, dichloromethane and TEA at 263k-273k. After stirring for 3 h, the solvent was evaporated and a light yellow oily substance was obtained by silica gel column chromatography. The light yellow oily substance was then dissloved in a mixture of acetonitrile, TEA and 1-tosylpiperazine. After stirring for 5 h, the title compound was obtained by silica gel column chromatography. Crystallization of the resultingg white solid from methanol afforded white crystals suitble for X-ray analysis.

### Refinement

The H atoms were positioned geometrioncally and refined using a riding model with d(C—H)=0.95–0.99 Å, and *U*_iso_(H)=1.2*U*_eq_(CH and CH2) or 1.5*U*_eq_(CH3) of the parent atom.

### Crystal data

**Table:**

C_20_H_25_N_3_O_3_S_2_	*F*(000) = 1776
*M**_r_* = 419.55	*D*_x_ = 1.372 Mg m^−^^3^
Orthorhombic, *P**b**c**a*	Mo *K*α radiation, λ = 0.71073 Å
Hall symbol: -P 2ac 2ab	Cell parameters from 12748 reflections
*a* = 13.062 (2) Å	θ = 1.7–28.0°
*b* = 15.710 (3) Å	µ = 0.29 mm^−^^1^
*c* = 19.798 (3) Å	*T* = 113 K
*V* = 4062.8 (11) Å^3^	Prism, colorless
*Z* = 8	0.24 × 0.20 × 0.18 mm

### Data collection

**Table:**

Rigaku Saturn CCD area-detector diffractometer	4844 independent reflections
Radiation source: rotating anode	4463 reflections with *I* > 2σ(*I*)
multilayer	*R*_int_ = 0.054
Detector resolution: 14.63 pixels mm^-1^	θ_max_ = 27.9°, θ_min_ = 2.1°
ω and φ scans	*h* = −17→17
Absorption correction: multi-scan *CrystalClear*	*k* = −20→20
*T*_min_ = 0.934, *T*_max_ = 0.950	*l* = −26→25
49454 measured reflections	

### Refinement

**Table:**

Refinement on *F*^2^	Primary atom site location: structure-invariant direct methods
Least-squares matrix: full	Secondary atom site location: difference Fourier map
*R*[*F*^2^ > 2σ(*F*^2^)] = 0.054	Hydrogen site location: inferred from neighbouring sites
*wR*(*F*^2^) = 0.143	H-atom parameters constrained
*S* = 1.14	*w* = 1/[σ^2^(*F*_o_^2^) + (0.0665*P*)^2^ + 2.3859*P*] where *P* = (*F*_o_^2^ + 2*F*_c_^2^)/3
4844 reflections	(Δ/σ)_max_ = 0.002
254 parameters	Δρ_max_ = 1.17 e Å^−^^3^
0 restraints	Δρ_min_ = −0.34 e Å^−^^3^

### Special details

**Table:**

Geometry. All e.s.d.'s (except the e.s.d. in the dihedral angle between two l.s. planes) are estimated using the full covariance matrix. The cell e.s.d.'s are taken into account individually in the estimation of e.s.d.'s in distances, angles and torsion angles; correlations between e.s.d.'s in cell parameters are only used when they are defined by crystal symmetry. An approximate (isotropic) treatment of cell e.s.d.'s is used for estimating e.s.d.'s involving l.s. planes.
Refinement. Refinement of *F*^2^ against ALL reflections. The weighted *R*-factor *wR* and goodness of fit *S* are based on *F*^2^, conventional *R*-factors *R* are based on *F*, with *F* set to zero for negative *F*^2^. The threshold expression of *F*^2^ > σ(*F*^2^) is used only for calculating *R*-factors(gt) *etc*. and is not relevant to the choice of reflections for refinement. *R*-factors based on *F*^2^ are statistically about twice as large as those based on *F*, and *R*- factors based on ALL data will be even larger.

### Fractional atomic coordinates and isotropic or equivalent isotropic displacement parameters (Å^2^)

**Table:**

	*x*	*y*	*z*	*U*_iso_*/*U*_eq_	
S1	−0.12585 (4)	0.69571 (4)	0.50761 (3)	0.03055 (16)	
S2	0.51658 (4)	1.09372 (3)	0.30610 (3)	0.03065 (16)	
O1	0.13122 (12)	1.01184 (10)	0.57588 (8)	0.0348 (4)	
O2	0.58272 (13)	1.14367 (10)	0.34791 (9)	0.0400 (4)	
O3	0.48630 (14)	1.12484 (11)	0.24122 (9)	0.0420 (4)	
N1	0.12333 (13)	0.89002 (11)	0.51535 (9)	0.0273 (4)	
N2	0.23688 (12)	1.01546 (10)	0.41984 (8)	0.0244 (4)	
N3	0.41057 (13)	1.07905 (11)	0.34905 (9)	0.0271 (4)	
C1	−0.11495 (17)	0.69019 (14)	0.59285 (13)	0.0329 (5)	
H1	−0.1601	0.6579	0.6204	0.039*	
C2	−0.03617 (16)	0.73600 (13)	0.61798 (11)	0.0293 (4)	
H2	−0.0186	0.7390	0.6645	0.035*	
C3	0.01696 (15)	0.77916 (12)	0.56530 (10)	0.0240 (4)	
C4	0.10712 (17)	0.83828 (14)	0.57598 (11)	0.0306 (5)	
H4A	0.0937	0.8758	0.6152	0.037*	
H4B	0.1694	0.8045	0.5857	0.037*	
C5	0.11965 (16)	0.84157 (14)	0.45193 (11)	0.0278 (4)	
H5A	0.1705	0.7949	0.4538	0.033*	
H5B	0.1384	0.8795	0.4139	0.033*	
C6	0.01385 (16)	0.80409 (14)	0.43904 (10)	0.0277 (4)	
H6A	−0.0342	0.8495	0.4249	0.033*	
H6B	0.0174	0.7610	0.4026	0.033*	
C7	−0.02224 (15)	0.76393 (13)	0.50316 (11)	0.0252 (4)	
C8	0.13226 (14)	0.97516 (13)	0.52092 (11)	0.0266 (4)	
C9	0.13997 (15)	1.02709 (14)	0.45566 (12)	0.0302 (5)	
H9A	0.0831	1.0106	0.4253	0.036*	
H9B	0.1316	1.0882	0.4667	0.036*	
C10	0.32177 (15)	1.05628 (13)	0.45626 (11)	0.0260 (4)	
H10A	0.3281	1.0309	0.5018	0.031*	
H10B	0.3072	1.1177	0.4617	0.031*	
C11	0.42175 (15)	1.04500 (14)	0.41822 (10)	0.0264 (4)	
H11A	0.4774	1.0755	0.4420	0.032*	
H11B	0.4399	0.9839	0.4163	0.032*	
C12	0.32501 (17)	1.03841 (14)	0.31276 (11)	0.0306 (5)	
H12A	0.3378	0.9766	0.3081	0.037*	
H12B	0.3183	1.0632	0.2670	0.037*	
C13	0.22808 (17)	1.05332 (15)	0.35261 (11)	0.0321 (5)	
H13A	0.2155	1.1152	0.3568	0.039*	
H13B	0.1693	1.0278	0.3285	0.039*	
C14	0.57571 (17)	0.99377 (13)	0.29458 (10)	0.0273 (4)	
C15	0.66141 (17)	0.97308 (14)	0.33228 (11)	0.0305 (5)	
H15	0.6860	1.0112	0.3658	0.037*	
C16	0.71122 (18)	0.89631 (15)	0.32094 (12)	0.0344 (5)	
H16	0.7705	0.8825	0.3465	0.041*	
C17	0.67544 (18)	0.83934 (14)	0.27257 (11)	0.0335 (5)	
C18	0.58779 (19)	0.86056 (14)	0.23633 (11)	0.0334 (5)	
H18	0.5617	0.8215	0.2040	0.040*	
C19	0.53773 (18)	0.93727 (14)	0.24632 (11)	0.0304 (5)	
H19	0.4785	0.9512	0.2207	0.037*	
C20	0.7307 (2)	0.75663 (16)	0.25933 (14)	0.0474 (6)	
H20A	0.7981	0.7581	0.2810	0.071*	
H20B	0.7392	0.7488	0.2105	0.071*	
H20C	0.6907	0.7093	0.2778	0.071*	

### Atomic displacement parameters (Å^2^)

**Table:**

	*U*^11^	*U*^22^	*U*^33^	*U*^12^	*U*^13^	*U*^23^
S1	0.0261 (3)	0.0310 (3)	0.0345 (3)	−0.0073 (2)	0.0029 (2)	−0.0032 (2)
S2	0.0320 (3)	0.0252 (3)	0.0348 (3)	−0.0070 (2)	0.0075 (2)	−0.0003 (2)
O1	0.0353 (9)	0.0313 (8)	0.0377 (9)	−0.0053 (7)	−0.0024 (7)	−0.0032 (7)
O2	0.0348 (9)	0.0328 (8)	0.0523 (10)	−0.0150 (7)	0.0129 (7)	−0.0125 (7)
O3	0.0482 (10)	0.0385 (9)	0.0391 (9)	−0.0008 (8)	0.0106 (8)	0.0138 (8)
N1	0.0287 (9)	0.0259 (9)	0.0274 (9)	−0.0068 (7)	0.0013 (7)	0.0031 (7)
N2	0.0197 (8)	0.0239 (8)	0.0295 (9)	−0.0049 (6)	−0.0029 (6)	0.0043 (7)
N3	0.0251 (9)	0.0267 (9)	0.0296 (9)	−0.0061 (7)	0.0007 (7)	0.0000 (7)
C1	0.0266 (11)	0.0255 (10)	0.0466 (13)	0.0001 (8)	0.0003 (9)	−0.0044 (9)
C2	0.0326 (11)	0.0271 (10)	0.0281 (11)	0.0028 (9)	0.0004 (8)	0.0047 (8)
C3	0.0261 (10)	0.0202 (9)	0.0258 (10)	0.0021 (7)	0.0017 (8)	0.0020 (7)
C4	0.0348 (11)	0.0304 (11)	0.0266 (11)	−0.0048 (9)	−0.0019 (8)	0.0035 (9)
C5	0.0266 (10)	0.0281 (10)	0.0287 (11)	−0.0057 (8)	0.0007 (8)	0.0012 (8)
C6	0.0290 (10)	0.0304 (11)	0.0238 (10)	−0.0041 (8)	0.0012 (8)	−0.0015 (8)
C7	0.0246 (10)	0.0219 (9)	0.0290 (10)	0.0001 (8)	0.0009 (8)	−0.0023 (8)
C8	0.0159 (9)	0.0266 (10)	0.0372 (12)	−0.0027 (7)	−0.0010 (8)	0.0012 (9)
C9	0.0197 (9)	0.0280 (10)	0.0428 (13)	−0.0018 (8)	−0.0005 (8)	0.0067 (9)
C10	0.0225 (9)	0.0252 (9)	0.0302 (10)	−0.0060 (8)	−0.0009 (8)	−0.0014 (8)
C11	0.0219 (9)	0.0288 (10)	0.0286 (10)	−0.0053 (8)	−0.0025 (8)	−0.0017 (8)
C12	0.0328 (11)	0.0309 (11)	0.0282 (11)	−0.0097 (9)	−0.0051 (8)	0.0040 (8)
C13	0.0269 (10)	0.0340 (11)	0.0356 (12)	−0.0071 (9)	−0.0075 (9)	0.0110 (9)
C14	0.0315 (11)	0.0273 (10)	0.0231 (10)	−0.0076 (8)	0.0059 (8)	−0.0021 (8)
C15	0.0307 (11)	0.0356 (11)	0.0251 (10)	−0.0085 (9)	0.0022 (8)	−0.0038 (9)
C16	0.0311 (11)	0.0380 (12)	0.0342 (12)	−0.0022 (9)	0.0022 (9)	0.0020 (9)
C17	0.0385 (12)	0.0306 (11)	0.0316 (11)	−0.0045 (9)	0.0118 (9)	0.0007 (9)
C18	0.0467 (13)	0.0304 (11)	0.0230 (10)	−0.0113 (10)	0.0065 (9)	−0.0045 (8)
C19	0.0355 (11)	0.0332 (11)	0.0225 (10)	−0.0075 (9)	−0.0003 (8)	0.0002 (8)
C20	0.0517 (16)	0.0343 (12)	0.0561 (16)	0.0025 (11)	0.0162 (13)	−0.0035 (12)

### Geometric parameters (Å, °)

**Table:**

S1—C1	1.696 (3)	C6—H6B	0.9900
S1—C7	1.729 (2)	C8—C9	1.531 (3)
S2—O3	1.4302 (18)	C9—H9A	0.9900
S2—O2	1.4309 (17)	C9—H9B	0.9900
S2—N3	1.6412 (18)	C10—C11	1.518 (3)
S2—C14	1.765 (2)	C10—H10A	0.9900
O1—C8	1.231 (3)	C10—H10B	0.9900
N1—C8	1.347 (3)	C11—H11A	0.9900
N1—C4	1.465 (3)	C11—H11B	0.9900
N1—C5	1.469 (3)	C12—C13	1.510 (3)
N2—C9	1.462 (3)	C12—H12A	0.9900
N2—C13	1.463 (3)	C12—H12B	0.9900
N2—C10	1.470 (2)	C13—H13A	0.9900
N3—C12	1.474 (3)	C13—H13B	0.9900
N3—C11	1.478 (3)	C14—C15	1.384 (3)
C1—C2	1.351 (3)	C14—C19	1.395 (3)
C1—H1	0.9500	C15—C16	1.389 (3)
C2—C3	1.424 (3)	C15—H15	0.9500
C2—H2	0.9500	C16—C17	1.392 (3)
C3—C7	1.354 (3)	C16—H16	0.9500
C3—C4	1.515 (3)	C17—C18	1.392 (3)
C4—H4A	0.9900	C17—C20	1.510 (3)
C4—H4B	0.9900	C18—C19	1.385 (3)
C5—C6	1.524 (3)	C18—H18	0.9500
C5—H5A	0.9900	C19—H19	0.9500
C5—H5B	0.9900	C20—H20A	0.9800
C6—C7	1.494 (3)	C20—H20B	0.9800
C6—H6A	0.9900	C20—H20C	0.9800
			
C1—S1—C7	90.96 (11)	N2—C9—H9B	108.9
O3—S2—O2	119.94 (11)	C8—C9—H9B	108.9
O3—S2—N3	106.26 (10)	H9A—C9—H9B	107.7
O2—S2—N3	106.67 (9)	N2—C10—C11	110.78 (16)
O3—S2—C14	108.00 (10)	N2—C10—H10A	109.5
O2—S2—C14	107.37 (11)	C11—C10—H10A	109.5
N3—S2—C14	108.14 (9)	N2—C10—H10B	109.5
C8—N1—C4	119.75 (18)	C11—C10—H10B	109.5
C8—N1—C5	125.96 (18)	H10A—C10—H10B	108.1
C4—N1—C5	114.08 (17)	N3—C11—C10	109.42 (17)
C9—N2—C13	108.82 (16)	N3—C11—H11A	109.8
C9—N2—C10	111.13 (16)	C10—C11—H11A	109.8
C13—N2—C10	109.16 (15)	N3—C11—H11B	109.8
C12—N3—C11	111.71 (16)	C10—C11—H11B	109.8
C12—N3—S2	116.61 (14)	H11A—C11—H11B	108.2
C11—N3—S2	116.59 (14)	N3—C12—C13	108.29 (18)
C2—C1—S1	113.78 (18)	N3—C12—H12A	110.0
C2—C1—H1	123.1	C13—C12—H12A	110.0
S1—C1—H1	123.1	N3—C12—H12B	110.0
C1—C2—C3	110.8 (2)	C13—C12—H12B	110.0
C1—C2—H2	124.6	H12A—C12—H12B	108.4
C3—C2—H2	124.6	N2—C13—C12	110.27 (17)
C7—C3—C2	113.39 (19)	N2—C13—H13A	109.6
C7—C3—C4	121.96 (18)	C12—C13—H13A	109.6
C2—C3—C4	124.64 (18)	N2—C13—H13B	109.6
N1—C4—C3	109.76 (17)	C12—C13—H13B	109.6
N1—C4—H4A	109.7	H13A—C13—H13B	108.1
C3—C4—H4A	109.7	C15—C14—C19	120.5 (2)
N1—C4—H4B	109.7	C15—C14—S2	119.55 (16)
C3—C4—H4B	109.7	C19—C14—S2	119.93 (18)
H4A—C4—H4B	108.2	C14—C15—C16	119.7 (2)
N1—C5—C6	111.90 (17)	C14—C15—H15	120.1
N1—C5—H5A	109.2	C16—C15—H15	120.1
C6—C5—H5A	109.2	C15—C16—C17	120.8 (2)
N1—C5—H5B	109.2	C15—C16—H16	119.6
C6—C5—H5B	109.2	C17—C16—H16	119.6
H5A—C5—H5B	107.9	C16—C17—C18	118.5 (2)
C7—C6—C5	107.88 (17)	C16—C17—C20	120.8 (2)
C7—C6—H6A	110.1	C18—C17—C20	120.7 (2)
C5—C6—H6A	110.1	C19—C18—C17	121.5 (2)
C7—C6—H6B	110.1	C19—C18—H18	119.2
C5—C6—H6B	110.1	C17—C18—H18	119.2
H6A—C6—H6B	108.4	C18—C19—C14	118.9 (2)
C3—C7—C6	125.32 (19)	C18—C19—H19	120.5
C3—C7—S1	111.06 (16)	C14—C19—H19	120.5
C6—C7—S1	123.52 (15)	C17—C20—H20A	109.5
O1—C8—N1	122.4 (2)	C17—C20—H20B	109.5
O1—C8—C9	119.81 (19)	H20A—C20—H20B	109.5
N1—C8—C9	117.75 (19)	C17—C20—H20C	109.5
N2—C9—C8	113.56 (17)	H20A—C20—H20C	109.5
N2—C9—H9A	108.9	H20B—C20—H20C	109.5
C8—C9—H9A	108.9		
			
O3—S2—N3—C12	43.28 (18)	C10—N2—C9—C8	−71.2 (2)
O2—S2—N3—C12	172.33 (16)	O1—C8—C9—N2	112.0 (2)
C14—S2—N3—C12	−72.46 (17)	N1—C8—C9—N2	−70.2 (2)
O3—S2—N3—C11	178.81 (15)	C9—N2—C10—C11	−179.18 (16)
O2—S2—N3—C11	−52.14 (17)	C13—N2—C10—C11	−59.1 (2)
C14—S2—N3—C11	63.07 (16)	C12—N3—C11—C10	−56.6 (2)
C7—S1—C1—C2	−1.04 (18)	S2—N3—C11—C10	165.84 (13)
S1—C1—C2—C3	1.2 (2)	N2—C10—C11—N3	56.3 (2)
C1—C2—C3—C7	−0.7 (3)	C11—N3—C12—C13	58.5 (2)
C1—C2—C3—C4	178.0 (2)	S2—N3—C12—C13	−163.93 (14)
C8—N1—C4—C3	129.51 (19)	C9—N2—C13—C12	−177.01 (17)
C5—N1—C4—C3	−45.7 (2)	C10—N2—C13—C12	61.5 (2)
C7—C3—C4—N1	15.1 (3)	N3—C12—C13—N2	−60.7 (2)
C2—C3—C4—N1	−163.44 (19)	O3—S2—C14—C15	140.22 (17)
C8—N1—C5—C6	−110.0 (2)	O2—S2—C14—C15	9.6 (2)
C4—N1—C5—C6	64.8 (2)	N3—S2—C14—C15	−105.18 (18)
N1—C5—C6—C7	−46.2 (2)	O3—S2—C14—C19	−37.9 (2)
C2—C3—C7—C6	176.41 (19)	O2—S2—C14—C19	−168.58 (16)
C4—C3—C7—C6	−2.3 (3)	N3—S2—C14—C19	76.67 (18)
C2—C3—C7—S1	−0.1 (2)	C19—C14—C15—C16	1.4 (3)
C4—C3—C7—S1	−178.81 (16)	S2—C14—C15—C16	−176.74 (16)
C5—C6—C7—C3	17.4 (3)	C14—C15—C16—C17	−0.7 (3)
C5—C6—C7—S1	−166.53 (15)	C15—C16—C17—C18	−0.8 (3)
C1—S1—C7—C3	0.62 (17)	C15—C16—C17—C20	178.8 (2)
C1—S1—C7—C6	−175.95 (18)	C16—C17—C18—C19	1.6 (3)
C4—N1—C8—O1	2.8 (3)	C20—C17—C18—C19	−178.0 (2)
C5—N1—C8—O1	177.38 (19)	C17—C18—C19—C14	−0.9 (3)
C4—N1—C8—C9	−174.92 (17)	C15—C14—C19—C18	−0.6 (3)
C5—N1—C8—C9	−0.4 (3)	S2—C14—C19—C18	177.51 (16)
C13—N2—C9—C8	168.59 (18)		

### Hydrogen-bond geometry (Å, °)

**Table:**

*D*—H···*A*	*D*—H	H···*A*	*D*···*A*	*D*—H···*A*
C5—H5A···S1^i^	0.99	2.77	3.469 (2)	128.
C6—H6A···O1^ii^	0.99	2.52	3.470 (3)	161.
C6—H6B···O2^iii^	0.99	2.51	3.346 (3)	143.

Symmetry codes: (i) *x*+1/2, −*y*+3/2, −*z*+1; (ii) −*x*, −*y*+2, −*z*+1; (iii) −*x*+1/2, *y*−1/2, *z*.

## References

[bb1] Cattaneo, M. (2009). *J. Thromb. Haemost.* **7**, Suppl. 1, 262–265. 10.1111/j.1538-7836.2009.03382.x 19630813

[bb2] Liu, D. K., Liu, Y., Liu, M., Zhang, S. J., Cheng, D., Jin, L. Y., Xu, W. R. & Liu, C. X. (2008). CN Patent 101284838A.

[bb3] Rigaku/MSC (2005). *CrystalClear* and *CrystalStructure* Rigaku/MSC Inc., The Woodlands, Texas, USA.

[bb4] Sheldrick, G. M. (2008). *Acta Cryst.* A**64**, 112–122. 10.1107/S0108767307043930 18156677

[bb5] Wallentin, L. (2009). *Eur. Heart J* **30**, 1964–1977. 10.1093/eurheartj/ehp296 19633016

[bb6] Zhi, S., Mu, S., Liu, Y. & Liu, D.-K. (2011). *Acta Cryst.* E**67**, o1490. 10.1107/S1600536811017107 PMC3120429 21754858

